# Critical evaluation of the JDO API for the persistence and portability requirements of complex biological databases

**DOI:** 10.1186/1471-2105-6-5

**Published:** 2005-01-10

**Authors:** Marko Srdanovic, Ulf Schenk, Michael Schwieger, Fabien Campagne

**Affiliations:** 1 Weill Cornell Medical College, 1300 York Ave, New York, NY 10021 USA; 2 FastObjects, Inc. 165 North Redwood Drive, Suite 200, San Rafael, CA 94903, USA

## Abstract

**Background:**

Complex biological database systems have become key computational tools used daily by scientists and researchers. Many of these systems must be capable of executing on multiple different hardware and software configurations and are also often made available to users via the Internet. We have used the Java Data Object (JDO) persistence technology to develop the database layer of such a system known as the SigPath information management system. SigPath is an example of a complex biological database that needs to store various types of information connected by many relationships.

**Results:**

Using this system as an example, we perform a critical evaluation of current JDO technology; discuss the suitability of the JDO standard to achieve portability, scalability and performance. We show that JDO supports portability of the SigPath system from a relational database backend to an object database backend and achieves acceptable scalability. To answer the performance question, we have created the SigPath JDO application benchmark that we distribute under the Gnu General Public License. This benchmark can be used as an example of using JDO technology to create a complex biological database and makes it possible for vendors and users of the technology to evaluate the performance of other JDO implementations for similar applications.

**Conclusions:**

The SigPath JDO benchmark and our discussion of JDO technology in the context of biological databases will be useful to bioinformaticians who design new complex biological databases and aim to create systems that can be ported easily to a variety of database backends.

## Background

Biological databases are key computational tools used daily by biologists. Such a large number of biological databases have been developed for biology that the Nucleic Acids Research Journal has published an annual database issue since 1996. From the point of view of the user, these resources are most useful when they are regularly updated and when they provide user-friendly ways to browse, search and view information. These user needs are generally recognized as important requirements by the designers and developers of biological databases. To cope with these requirements, bioinformaticians who develop the biological databases have typically responded by developing increasingly customized software to manage the data and the information (e.g., [1-4]). In doing so, and to facilitate the software development effort needed to create a biological database, bioinformaticians have used a variety of information technologies. These technologies range from the ones that make it possible to create dynamic web applications (e.g., Common Gateway Interface/CGI, Java Servlets, web application frameworks), to technologies needed to store the data and the information in a persistent manner (i.e., text files [5,6], relational databases [7], frame representation systems [8-10], object-oriented databases [11]).

In this article, we report on our experience with the Java Data Objects persistence technology and take a critical view at the advantages and drawbacks of this emerging Java persistence standard for the development of advanced biological databases. We have ported the SigPath information management system (see below) to the JDO API and have defined an application-specific benchmark. We used this benchmark to evaluate the performance of two JDO implementations that target either a relational or an object database backend. This article summarizes the performance results that we obtained, announces the availability of the SigPath JDO benchmark (available under the GPL license), and identifies areas where the JDO API could be refined to facilitate portability and scalability of applications.

### Data persistence

Biological databases are built with software that executes on computers. Most biological databases are of a size that could fit entirely in the central memory of modern computers. However, because computers may need to be shutdown for maintenance – or may crash inadvertently – data for a given database cannot be kept in computer memory for the life of a biological database. This problem is not specific to biological databases so that a variety of data persistence approaches and technologies are available. The key role of these technologies is to guarantee that data persists safely between the invocations of the programs that may modify the data.

### The pros and cons- of persistence technologies for biological databases

Data can be stored in **text files with limited structure **and important information can be stored in unstructured text files expressed in English. Unstructured flat-files do not help perform large-scale analyses, structured queries or integrate data across multiple sources, all of which are important requirements for biological databases. Therefore, unstructured files are now widely recognized throughout the field as inadequate for the management of biological information.

**Highly structured data formats**, such as ASN.1 [12] and more recently XML, are a more favored alternative. They can support structured queries, large scale analyses and data federation. Structured file formats, however, do not provide support for concurrent manipulation of the information by several users (e.g., several curators interacting with a submission tool to input new data about one protein in the database). As such, they are adequate for data exchange among systems, but not for concurrent access. Since the file format offers no support for synchronization, locking or complex domain-dependent data validation rules (for XML, XML Schemas are limited to simple validation rules), using structured data formats for biological information storage forces system developers to implement these services explicitly as a layer between the business code and the data storage. For instance, since XML Schemas are not capable of validating XML data with respect to information outside of the scope of the file being validated (such as data in other files or in a database), developers must implement custom validation code. XML Schema focus on syntactic validation, while most applications require semantic validation [13].

**Database management systems **(DBMS) have been historically developed to abstract the services (such as synchronization, business domain constraints) needed by systems that need to support large number of users accessing a shared storage of data. A few types of DBMS exist that differ in the way they represent data. Relational DBMS represent data as tables that contain rows and columns of various types, while Object DBMS support the concept of object classes and object instances directly.

**Relational DBMS **such as Postgres, MySQL or Oracle have been used to store biological information in many laboratories, including ours [14,15]. A short introduction to using RDBMS for biological information storage was recently offered in [16]. Briefly, complex relationships among elements of information are stored in relational databases by expressing relations among records in several tables. The technology is useful for a variety of biological databases, where the mapping between the biological data and the relational data model is simple.

However, the technology has two major drawbacks for advanced biological databases. The first problem is because of a mismatch between the object-oriented programming style and the relational data model. Advanced biological databases often require programs that manipulate tens or hundreds of object classes. Data in the instances of these classes needs to be made persistent, and this requires writing mapping code. The mapping code takes a graph of objects and transfers the data in this graph into records in the various tables of the relational database. Mapping code needs to be developed for the reciprocal operation, from the relational records to the object instance graph. Depending on the complexity of the relationships among objects in the graph, the development of the mapping code may represent a significant part of the code developed for the overall database.

**Object DBMS **such as O2 [17] and FastObjects have been developed to eliminate the need to write mapping code, and to store objects directly in native form in the database. This approach was reported to offer substantial performance improvements and reduced development and maintenance costs for data organized in an object graph with complex relationships.

### Java Data Objects Technology

The Java Data Objects Technology (JDO) is a Java application programming interface (API). This API was developed as a Java Specification Request [18] to offer: "a standard way to store Java objects persistently in transactional data stores..., a standard way to treat relational database data as Java objects, and a standard way to define transactional semantics associated with those objects."

JDO appears as an attractive technology for the development of biological databases for the following main reasons:

1. It is designed to offer portability across a wide range of transactional stores or database backends, from open-source relational databases to native object oriented databases.

2. It transparently handles object persistence when relational or object persistence backends are used (the developer only manipulates objects and classes and does not need to write mapping code).

3. JDO also handles persistence transparently for object oriented databases, where mapping code is not needed.

4. It is a Java technology that integrates seamlessly with web application servers (e.g., Tomcat, JBoss, etc.) often used to create the web front-ends of a biological database.

### A critical evaluation of the JDO technology

Given the stated advantages of the technology we decided to carry out a critical evaluation of JDO to determine if the technology can routinely be used for the development of advanced biological databases. Our evaluation focused on the following questions:

**Portability: **Is JDO a mature API that can guarantee portability of the application across database backends?

**Performance: **If portability is achieved, how do relational and pure object oriented backends compare in term of performance?

**Biological database specific requirements: **Do complex biological databases have specific requirements that JDO 1.0.1 does not address?

To answer these questions, we have ported a biological information management system (the SigPath system, see below) to the JDO 1.0.1 API. (The SigPath system was originally implemented with the ODMG API [19]). In the first step of the port, we compiled the new code with the FastObjects JDO implementation [20] FastObjects JDO is an implementation of the JDO API that connects to the native FastObjects object database. In a second step, we have adapted the existing code to support exchanging the JDO implementation and database backend between the FastObjects implementation and the Solarmetric Kodo implementation of JDO [21]. Kodo is an implementation of JDO 1.0.1 that connects to a variety of relational database backends. The aim of the second development was to modify the code to make it possible to switch from FastObjects JDO to Kodo JDO by changing a configuration property, and then simply recompiling. Our aim was to create a code-base that was fully portable from a relational database backend to an object-oriented database backend to address the portability question.

## The SigPath Information Management System

SigPath is an open-source project aimed to develop an Information Management System (IMS) to foster modeling and simulation of cell signaling pathways and networks (see the SigPath project [22]) [23]. The SigPath IMS appears to the end-user as a web application that provides search, browsing and visualization capabilities. The project home page provides tutorials that explain how the system is typically used.

Most traditional biological databases focus on one type of database entry (e.g., gene, mRNA, protein, protein motif, protein domain, etc.) and store information in database entries. This approach has been very useful to create detailed catalogs of biological parts and is a critical and essential element of the bioinformatics resources that support modern biological research. However, certain integrative studies, such as systems biology and modeling and simulation of biochemical pathways call for databases that integrate several types of information.

**Figure 1:**
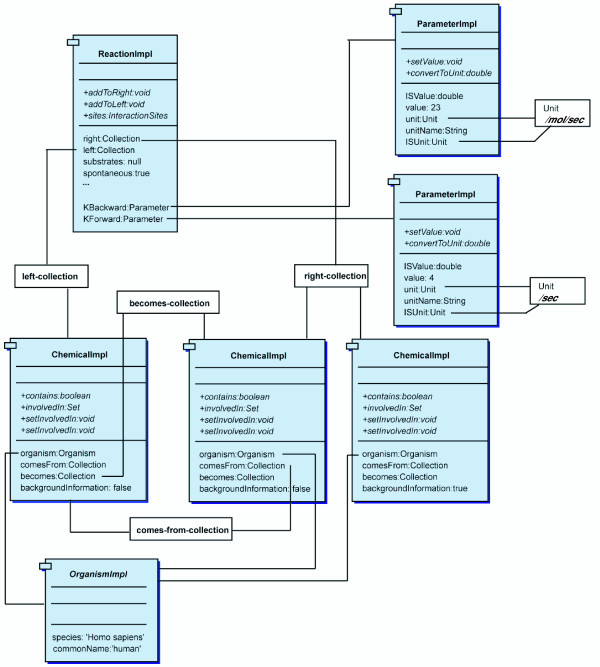
**Instance graph for representation of a reaction between three chemicals (e.g., *A *↔ *B *+ *C*)**. Some of the relationships that support navigation from reactions to chemicals are shown, as well as links between chemicals. The three chemicals are produced by the "human" organism. Quantitative kinetic parameters are also shown for the reaction (backward and forward rate of the reaction). The figure illustrates how a graph of object instances is used to represent biological information corresponding to a biochemical reaction.

The SigPath IMS is an example of an advanced biological database that encodes information through a number of information types and a set of relationships among them. Figure 1 illustrates how SigPath encodes information about a biochemical reaction: the reaction is represented as a graph of object instances.

### Introduction to the SigPath ontology/database schema

**Figure 2:**
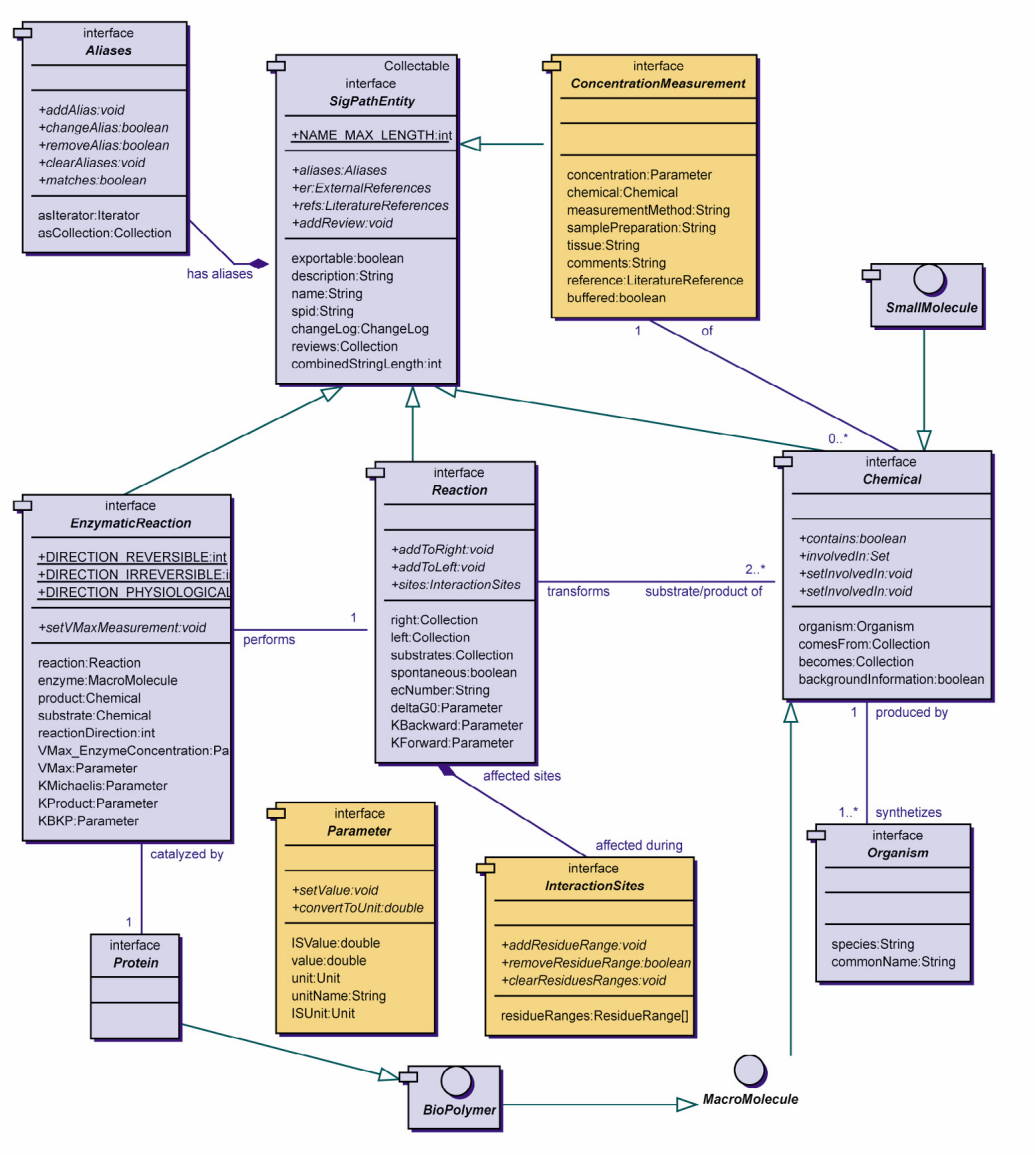
**Fragment of the UML diagram for the SigPath ontology/database schema**. The Figure shows how instances of certain classes are related and how the relationships among classes can be used to represent information. The link between Reaction and Chemical expresses the information that reactions transforms chemicals, and that chemical can be substrate or products of reactions. The classes and attribute marked in orange are specific to the SigPath ontology and have no equivalent in the EcoCyc ontology.

A fragment of the SigPath ontology is given on Figure 2 as a UML diagram. The description of the complete set of persistent classes used in SigPath is given on the project web site ([22], see the "for developers" tab).

The SigPath system supports several types of biological information, ranging from information to represent small molecules and proteins to the interactions between these molecules. The main information types supported by SigPath are listed on Table 1. In SigPath, information is represented in an object-oriented manner, with information types often associated with classes. The SigPath object-oriented database schema was adapted from the EcoCyc ontology [9]. Several classes presented on Table 1 have an equivalent in the EcoCyc ontology. In the rest of this article, we will use the terms ontology and JDO database schema indistinctively, as they represent very similar concepts: a class in the object-oriented schema of SigPath is equivalent to a frame in the EcoCyc ontology, and an attribute of an object class is similar to the slot of a frame.

This multiplicity of SigPath information types and the variety of relationships among them makes it important to clearly define what type of information can be represented by the system (i.e., the set of object graphs that could potentially be created and stored in the database). This information is formalized in the SigPath ontology. This ontology is implemented in a JDO database schema. (In the SigPath system, the set of allowed object graphs may be further reduced by adding semantic constraints to the validation mechanism used during information submission). The JDO schema consists of the set of SigPath Java classes that are persistent and of meta-data about these classes. Meta-data is expressed in JDO files and provides information about the classes that cannot be expressed directly in the Java language, for instance, type of the elements for the collection field of the persistent classes. Figure 5 shows a small JDO file and illustrates the type of information that it provides. A thorough presentation of the structure of JDO files is given in [24], vendor-specific extensions are documented in each JDO implementation.

The SigPath code base has specific characteristic that make it a useful resource for evaluating JDO technology:

• SigPath is an open-source project released under the GPL, so that the benchmark code is freely available for others to study, reproduce our results, or extend the benchmark to other JDO implementations or database backends.

• SigPath is both a web-based application and a batch-oriented application.

• The SigPath code-base includes unit tests [25] that help verify that the application behaves correctly against two different database backends.

• The SigPath system provides varied use cases that exercise different behaviors of the database backend and JDO implementation (see use cases below).

In the next section, we present the methods that we used to evaluate JDO technology for the creation of advanced biological databases.

## Results

This section describes the results of the SigPath JDO benchmark and addresses the portability and performance questions described in the introduction.

### SigPath: porting from one JDO implementation to another

We modified the FastObjects JDO version of SigPath to compile indifferently with the FastObjects and Kodo implementations of JDO. The modifications that we had to make to the project were (i) modifications to the JDO file, (ii) modifications of the code base and (iii) modification of the code base and application data.

#### Modifications to the JDO file

The Kodo enhancer tool performs stricter semantic validations on the JDO files than the FastObjects enhancer. Modifications needed to pass the validation tests were:

1. Added persistence-capable-superclass attribute to classes that have a persistence capable superclass. This attribute is optional for FastObjects, which uses Java reflection by default, but is strictly required by the Kodo implementation (in agreement with the JDO specification).

2. Removed all interfaces from the JDO file. Enhancing with Kodo failed when interfaces were listed in this file. Since FastObjects requires interfaces to be listed as persistent classes, the SigPath build script conditionally includes such statements in the JDO file when FastObjects is configured. The JDO specification does not mention interfaces, so that the behaviour of JDO implementation is left undefined.

3. (As a result of 2.) Replaced references to interfaces with references to implementation (e.g., replaced Protein by ProteinImpl) throughout the JDO file.

4. Added collection element types to all persistent collections. FastObjects requires the type to be specified when the collection is used in a query. Kodo requires the type to be defined for each collection, otherwise Kodo will try to serialize the collection and store it as a binary object. If the persistent class is not serializable, this mechanism will fail. Therefore, for this benchmark, we explicitly defined the collection types for each collection.

5. Removed field definitions from sub-classes when they refer to fields of a super-class. (e.g., the field "reactions" in Model was specified twice in the Model sub-class and in the Pathway super-class). Removing these duplicate declarations is consistent with the JDO specification.

Furthermore, the Kodo enhancer expects classes to be listed in the JDO file in a specific order. The enhancer fails if a class appears in the JDO file before another class that the first class references. Therefore, we reordered the class definitions in the JDO file. (We verified that this is no longer an issue with version 3.0 of Kodo, but keep this description as other JDO implementations may share the same limitation).

Finally, we added Kodo extensions to the JDO file to create indexes on the tables that were used extensively in queries. All changes to the JDO file were consistent with the JDO specification. Index tuning was performed by running the boot and test part of the benchmark and the small molecule import with various indexes choices.

#### Modifications to the code base

**Figure 3:**
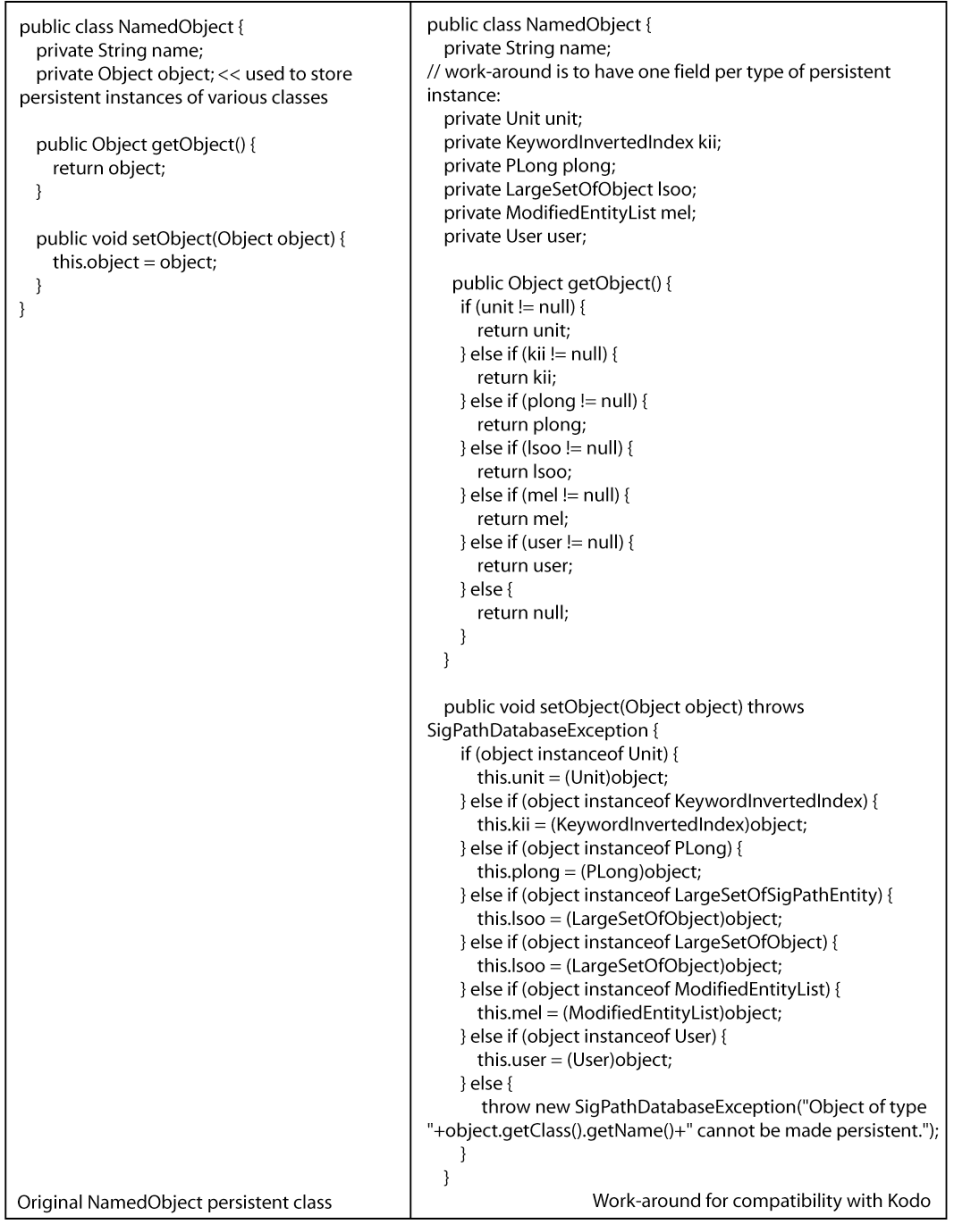
Work-around for classes that contain an object field.

We modified the code base to work-around a limitation of the Kodo implementation. With Kodo, instances of classes that contain java.lang.Object fields are made persistent with the object field stored as a BLOB in the database. Storing objects as BLOBs puts strong limitations on their use. For instance, it is impractical to query for these objects by their fields (e.g., querying directly for a User instance by the id of the user is not possible if the instance is stored as a BLOB). Storing such fields as BLOBS was therefore not acceptable for certain types of persistence objects, and we implemented the work-around shown on Figure 3.

**Figure 4:**
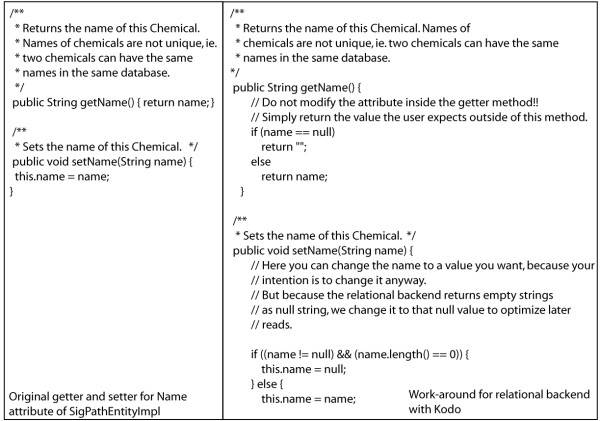
Work around for classes that have String getter and setters, when empty strings can be made persistent.

**Figure 5:**
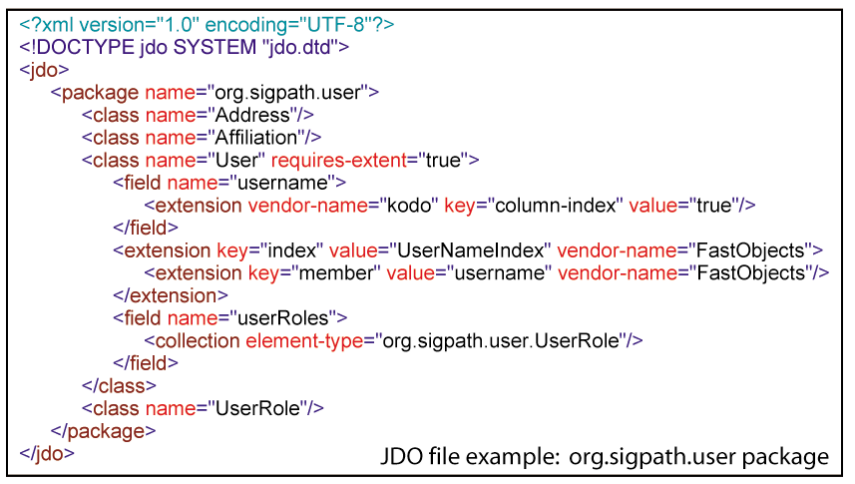
**Example of JDO file**. This file is used to define the persistent classes that are used in SigPath to represent end-users. Four persistent classes are shown: Address, Affiliation, User and UserRole. The <field> element can be used to refer to specific fields of persistent classes (such as the username field of class User on this example). The userRoles field is described to be a collection that contains elements of type UserRole. Elements called <extension> make it possible to provide vendor specific directives, such as to define indices on a persistent field.

Another code modification was required to work-around a problem with the database backend that did not handle appropriately empty strings (“”). The database backend used for this benchmark stored empty strings as null. Reading these strings back from the database resulted in null being obtained from Kodo instead of empty strings. This resulted in several unexpected NullPointerException being thrown during the JUnit tests. Figure 4 illustrates the approach that we used to work-around this problem.

#### Modification to the code base and benchmark/application data

Finally, we had to modify the code base to put a limit on the length of long strings. Using a relational database backends imposes to define the maximum length of each string attribute defined in the persistent classes of the application. For instance, a limit must be set on the name attribute of the SigPathEntityImpl shown on Figure 2. We initially used the default maximum length for all fields and found that certain fields could be longer than this limit when running the test and the benchmark. For instance, description fields of ProteinImpl are imported into SigPath from the DE line of SwissProt and TrEMBL entries. Some entries have long descriptions (that can exceed 1,000 characters). To test the impact of this limit on the code of the application, we arbitrarily choose to use a maximum length of 1,000 characters. We excluded from the benchmark input data proteins and small molecules that had aliases or descriptions longer than 1,000 characters, and other entries that would exceed any String field limit. This was done to make sure that the same input data was used for both the FastObjects and the JDO relational benchmarks.

### Performance measurements

A brief summary of the performance measurements obtained with the SigPath benchmark is given in Table 3. The table presents time measurements for each use case of the benchmark. The measurements are listed both for the FastObjects JDO implementation (columns marked FO) and for the Kodo implementation. Columns marked %FO/KODO indicate the percentage of the time running the benchmark with FastObjects takes compared to running the benchmark with Kodo. The last column of the table FO/KODO CV indicates the coefficient of variation of the total time across four independent measurements. Small values of CV (1–5%) indicate consistency between the four measurements. However, some use cases showed higher variations (10,11,12,36%), so we report as well the minimum value of the four time measurements for both FO and KODO (in columns marked Min).

The raw data used for the calculation of these performance measures is provided in the supplementary material and on the SigPath JDO benchmark pages. These pages also provide the logs from which the raw data has been collected.

## Discussion

### Portability

Our port of SigPath confirms that JDO greatly facilitates the porting of a bioinformatics application from one database backend to another. However, we report here several modifications that we had to make to the SigPath system to achieve this level of portability. This suggests that there is a need to develop JDO compliance tests that could be used to test that a specific implementation of a JDO-aware database is really compliant with the standard. This test suite would validate that JDO enhancers accept correct JDO files and correctly reject JDO files that break the specification. The differences in the interpretation of JDO files that we noticed between FastObjects and Kodo (see Results section) practically limit the portability of JDO applications. This article has presented techniques that can be used to work around these limitations until a JDO compliance test is developed and used. We note that the work arounds that we described may be specific to the two JDO implementations that we tested, and that other work arounds may be needed to achieve portability with other JDO compliant backends.

Surprisingly, we found that an outstanding portability problem is in the way the different JDO back-ends store long strings of characters. While the FastObjects backend put no limitation of the length of long strings, the relational back-end used with Kodo limited the length of long strings to 4,000 characters. This limit had to be chosen and set for each persistent string field used in the application (when the default value was not appropriate). Although 4,000 characters may appear a large limit, it is likely to be reached in bioinformatics application either with textual or with sequence data. When this happens, the application will have to be re-engineered to work around the fixed limit. A work-around could be to use a data type that does not have a length limitation, but these data types also have other limitations (for instance, usually indexes cannot be used on those fields). Whichever solution is chosen, this issue must be considered early during the design of the application. It would be useful if the JDO standard offered a mechanism for the application developers to specify which string length their application requires to function properly with JDO backends. Each enhancer could then check that the application is requesting a maximum string length that is compatible with the database backend and fail early if it does not. (As of now, these types of error will most likely be detected when testing the application.)

### Performance

The SigPath benchmark provides precise measurements of the performance of one biological database application against two JDO compliant database backends. The measurements were performed on use cases that are typical of the activities needed to develop the code of SigPath and to deploy a production SigPath system. (Table 2. indicates which use cases belong to our software development process and which belong to administrative and curation tasks that we need to carry out to prepare a new release of SigPath).

As shown in Table 3, performance varies widely with the type of use case, but is overall significantly better with the object database backend. Use cases that perform batch loading of protein information into the database benefited the most from using the native object database FastObjects backend (with loading of data sometimes completed five times faster than with Kodo and a commercial relational backend). An exception to this trend is the SM Import use case, which shows only a 3% performance difference. This use case reads an XML file and loads small molecules into the database. To do so, it checks for each molecule that the accession code of the new molecule does not already exist in the database (this is an error condition that would interrupt the import). Since the database does not contain small molecules, the query used to perform this check returns an empty set for each molecule of the import. It appears that this specific operation is slower with the object-oriented backend that we have used for the benchmark.

The last column of Table 3 indicates the coefficient of variation (CV) of the individual measurements (among four independent executions). The CV values indicate that the performance of certain steps vary significantly from execution to execution. These differences are likely to be caused by the caching behavior of the database server and of the operating system. Caching can occur because we have not restarted the database server between the benchmark runs, or rebooted the machines. These differences may also be caused to a lesser extent by variations in what operating processes were active and the amount of IO wait at the time that the specific use case was executed. We have tried to reduce such causes of variability (see methods) but have not attempted to eliminate them completely (e.g., setup an isolated database server and disable all interactive use of the server). Our rationale is that such variability, including caching, is representative of a typical production system. Given the CV, the average execution time may not be an accurate representation for some use cases, so we report also the minimum execution time across the four independent executions of the benchmark.

The benchmark provides an indication of how well an object-oriented database system performs compared to a relational database backend for the SigPath use cases. A known limitation of benchmarks is that the performance measure that they provide are specific to the application tested, and may not generalize well to other use cases. Also, the SigPath benchmark does not cover multithreaded/multiclient operations. Results may vary depending on the chosen locking strategy and the number of clients/threads running parallel. Given these caveats, however, this benchmark indicates that, for most of the SigPath use cases, the performance of the SigPath system is significantly improved when using a native object database system. Particularities of the SigPath benchmark that may correlate with this result are (i) the complexity of the database schema (75 persistent classes) and (ii) the number of connections that exist among instances of these various classes.

Finally, these results and our distribution of the SigPath benchmark source code can help vendors diagnose performance problems with their implementation of the JDO implementation, and provide users with an objective measure of the performance a given JDO implementation, for similar types of applications.

### Biological database specific requirements

During our evaluation of the JDO technology, we have noted that two common requirements of advanced biological databases are currently not being addressed by JDO.

#### Support for interfaces

**Figure 6:**
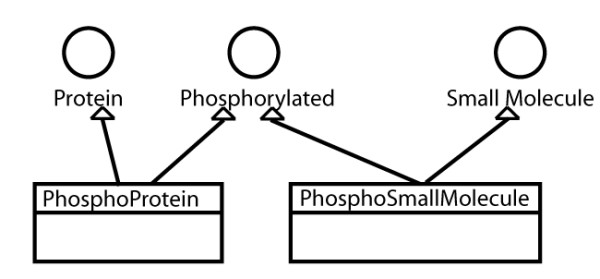
**Illustration of the use of interfaces to express is-a relationships among biological concepts**. Circles represent interfaces while boxes represent classes. An arrow from one class to an interface indicate that the class implement the interface, and this relationship can be used to indicate that instances of the class have the properties described by one or several interfaces.

When designing a biological database schema, it is often useful to express that one class shares the properties of two or more classes. In a programming language such as C++ this can be represented as multiple inheritance (one class inherits from two parents) while in a programming language such as Java, this concept is represented with interfaces (one class implements two interfaces). In the context of JDO, consider the class diagram shown on Figure 6. The diagram illustrates one way to represent the phosphorylated forms of protein and small molecules. On this diagram, one has represented a "Phosphorylated" interface which is implemented by PhosphoProtein and PhosphoSmallMolecule. While this way to represent biological information is useful, JDO does currently not specify the handling of interfaces, so that the design shown on Figure 6 can not be implemented with JDO in a portable way. (This design would work with FastObjects, but not with Kodo.) The JDO specification should clarify if interfaces must be supported by for a JDO implementation to be compliant with the standard.

#### Support for large number of objects

Biological databases often need to manage large number of objects (e.g., large number of proteins, small molecules, etc.). For instance, SigPath stores information about several hundred of thousands of proteins. We found that JDO 1.0.1 lacks some features that would facilitate writing scalable applications.

An example is that the JDO standard does not provide a scalable way to determine the number of persistent instances of a given class. The JDO compliant way to accomplish this operation is to obtain a reference to a collection of instances of this class (using a JDO Extent), and to call the size() method on this collection. Since the collection must first be obtained from the database server before the size() method can be invoked, this procedure takes a time proportional to the number of instances of this class. Most database backends store the number of instances of a certain class in the database and can determine this information in a constant time, so a standard way to obtain this information from a JDO implementation would be very helpful (SigPath can use either a pure JDO extent sizing method, or a vendor-specific method through an extension mechanism implemented in the source code, so that performance can be compared).

A second example is that JDO 1.0.1 does not provide support for queries that return large result sets. Under standard JDO 1.0.1 behavior, traversing a persistent collection (by accessing each element of the collection in turn) brings the entire contents of that collection into memory. This behavior is appropriate for small result sets. However, there are cases where the complete set of instances returned by a query cannot be processed within a single transaction. This occurs for instance when all the results returned by a query do not fit in the fixed memory limit allocated to the Java Virtual Machine. In such cases, it may be necessary to obtain the result of a query in chunks of a certain number of records/instances (for instance 1,000 or 10,000 instances at a time), and process them in independent transactions. Upon transaction commit, memory associated with a chunk is released and can be used to process the next chunk. Implementing this type of scalable processing in an efficient manner usually requires making modifications both in the persistent class of elements in the result set and in the query filter. The class of the elements in the result set can be modified to add an instance identifier that can be used both to sort the instances and to select only those within the current processing chunk. The query filter can be modified to add a clause that selects only instances of the next chunk, based on the identifier introduced in each element. An alternative is to provide an API call to notify the JDO implementation that instances which have been processed can be evicted from memory. Since several vendors already have their own extensions to provide scalability feature, it would be useful for JDO to support such features through a standard API.

## Conclusions

Here, we have shown that it is possible to develop a bioinformatics database that can be reconfigured automatically and recompiled to run either against a relational database backend or against an object database backend. The key advantage of this added flexibility is that the bioinformatics database becomes portable with respect to the database backend. This has important implications for the development of open-source bioinformatics databases. In such projects, usually more than one laboratory contributes to developing the software of a specific biological database. Therefore, it is useful if each laboratory can choose a database backend for development and deployment, yet contribute to the project in a shared code base.

The Java Data Objects standard offers the productivity gains of transparent object persistence, and a fine-grained object persistence model useful to represent many biological concepts. We discussed why JDO can appear as an attractive option for the development of advanced biological databases and the type of problems that we encountered when implementing and deploying a biological database against two different JDO implementations. The future JDO standard (JDO 2.0) should address some of the issues that we discussed in this article (e.g., support for interfaces, or for large result sets). When JDO 2 implementations become available, we expect that JDO technology will have a significant impact on the design of high-performance biological databases that need to represent and manage complex biological information types.

## Methods

### Benchmark use cases

To address the performance question, we have developed benchmark use cases. The benchmark use cases were designed to be representative of performance that one would observe when either (i) developing the software of the SigPath system or (ii) preparing a new release of the SigPath IMS (includes loading the database with information from other databases). Our benchmark thus considers both the development and the production stages of the life-cycle of the application.

The use cases, or benchmark steps and a summary of their purpose in the context of the SigPath project are listed on Table 2.

The boot and test steps make it possible for the SigPath developers to create a sample database and test that important functionalities of the application are working satisfactorily.

**boot **– The boot step compiles the sources of the project, enhances the JDO persistent classes (a program, called a JDO enhancer, transforms Java class files into persistent classes and allow them to interact with the JDO implementation), creates an empty database and imports information into the database. Importing this information involves parsing an XML file that contains the information, validating this file against the SigPath information exchange XML Schema, validating against additional semantic rules that cannot be expressed with XML Schemas (database lookups are used during this step to connect new instances to instances previously submitted in the database, if needed), and saving new persistent instances to the database. The boot sample data is designed to contain at least one instance of each type of information that can be stored in the SigPath IMS.

**test **– The test step runs JUnit tests against the data that was imported during the boot step. The JUnit tests assert that information stored in the database corresponds to the information in the boot XML file. For instance, the tests check that the number of persistent instances matches the number found in the boot import file, but also that specific elements of information have been saved accurately. Furthermore, the tests assert various semantic properties of the application and database access code, running queries against the database, navigating through objects, creating new persistent instances or deleting them, etc. The complete set of operations performed in the test is fully described in the source code for the JUnit tests (see edu.mssm.crover.sigpath.test package, and specifically the class MasterTest).

**small molecule import **– This step creates SmallMoleculeImpl persistent instances (implementation of the SmallMolecule interface shown on Figure 2). The data used to create these molecules is a modified form of the NCI open database. Only small molecules that have a name, description, aliases and SMILES representation are imported from NCI Open (the total number of molecules read is 237,771, and the total number of molecules loaded into the database is 45,229). These data are imported and stored in the attributes of SmallMoleculeImpl (most fields: name, description and aliases are inherited from SigPathEntityImpl).

**protein import **– This series of steps creates ProteinImpl persistent instances (implementation of the Protein interface, shown on Figure 2). The data to load these proteins is obtained from a simplified XML format created from SwissProt and TrEMBL data files with SwissKnife [26]. The exact list of files imported is given on Table 2.

**full text indexer **– The SigPath system offers users the ability to search entities by keywords. This step builds an inverted full text index [27] that is used by the web application to accelerate keyword-based queries. An inverted full text index links each keyword that occurs in text strings of a SigPath entity (e.g., name, description, aliases) to the SigPathEntity instance that contains the keyword. This step creates 465,679 Keyword instances that link to a total of 345,133 SigPath entities (small molecules or proteins).

**XML import **– This step is similar to the loading of SigPath information in the boot target, but loads information obtained from the online version of SigPath (this benchmark used the information as of October 14^th ^2003). For this benchmark, XML import instantiates 14 SmallMoleculeImpl, 8 ProteinImpl, 121 ComplexImpl, 77 modified chemicals (SmallMoleculeImpl or ProteinImpl), 92 ConcentrationMeasurement, 165 ReactionImpl, 75 EnzymaticReactionImpl, 23 Model, 3 Pathway and 27 PendingReviews.

### Benchmark procedure

The benchmarks were run as described on the SigPath Project web site ([??] see the "JDO benchmark" tab). Each benchmark (FastObjects or Solarmetric Kodo with a relational database) was run on a two Xeon 3GHz processor machine with hyper-threading on and 6 Gb of memory. The machine was running Red Hat Advanced Server Linux 2.4.21-4.0.1.ELsmp, and was used both as database server and database client (to minimize the impact of the network on performances). No significant other processes were running on the benchmark machine while the tests were executed. We benchmarked FastObjects t7 server version 9.0.7.185 and Kodo JDO version 2.5.3. Each benchmark was run four times to average the effect of variability in the computational environment that may not have been controlled by our benchmark procedure. The results report the coefficient of variations (mean divided by the standard deviation) of the total running time for each use case and this helps point out cases when the computational environment had an effect on measured times. We believe that these variations are common in a production environment and report the average total running time as well as the minimum total running time for each use case.

## Authors' contributions

Marko Srdanovic and US implemented significant components of the FastObjects and Kodo JDO ports. Marko Srdanovic collected benchmark data at WMC and US collected similar data at FastObjects. Michael Schwieger and FC designed the study and contributed to the JDO ports. FC drafted the manuscript. All authors read and approved the final manuscript.

## Figures and Tables

**Table 1:** Selected information types supported in SigPath.

**Classes**	**Description**
SmallMolecule	A small molecule, such as ATP
Complex	A binding complex between two or more molecules
Protein	A protein molecule
Chemical	A small molecule, protein or complex
Reaction	Reaction between molecules: has substrates and products
EnzymaticReaction	Associates a reaction to the enzyme that catalyze it
Unit	Represents a unit, such as mol/l or /sec
Parameter	Associates a value to its unit
ConcentrationMeasurement	Associates a concentration to a molecule and the conditions of the measurement
Pathway	A set of reactions and enzymatic reactions
Model	A quantitative biochemical model, a set of reactions (or enzymatic reactions), initial concentrations for molecules in the model, rates of the reaction, kinetic parameters of the enzymatic reactions.

**Table 2:** Overview of the use-cases in the SigPath Benchmark

**Project stage**	**Step**	**Utility for the SigPath project**
development	boot	Loads a sample set of information that can be used to run JUnit tests and for interactive testing of the web application
development	test	Performs JUnit tests to verify that key aspects of the system are working correctly
pre-release	SM import	Import small molecule information (names, aliases SMILES from NCI Open)
pre-release	Full Text Indexer 1	Builds an inverted full text index for small molecules imported in previous step
pre-release	mam.xml	Imports TREMBL mam.dat proteins
pre-release	rod.xml	Imports TREMBL hum.dat proteins
pre-release	hum.xml	Imports TREMBL inv.dat proteins
pre-release	inv.xml	Imports TREMBL inv.dat proteins
pre-release	vrt.xml	Imports TREMBL vrt.dat proteins
pre-release	sprot41_1.xml	import SwissProt 41.dat, part I
pre-release	sprot41_2.xml	import SwissProt 41.dat, part II
pre-release	Full Text Indexer 2	Builds an inverted full text index for the proteins imported from TrEMBL and SwissProt
pre-release	XML Import	Imports data from another SigPath database (data is encoded in the SigPath XML exchange format)
traversal simulation	TestGetPathways	Navigates through the Pathway instances. Used to simulate user navigation on the web site.
traversal simulation	TestBenchmark	Another benchmark-specific performance test.

**Table 3:** SigPath benchmark measurement summary.

**Minimum of four measurements**				
	**FO Min**	**KODO Min**	Δ **(KODO – FO)**	**%(FO/KODO) Min**

**Junit Tests**	7809	14962	7153	**52.19%**
**Boot**	3026	3976	950	**76.11%**
**SM Import**	399189	409452	10263	**97.49%**
**FullTextIndexer1**	566858	1804663	1237805	**31.41%**
**mam.xml**	58999	299876	240877	**19.67%**
**rod.xml**	95680	422423	326743	**22.65%**
**hum.xml**	144250	692476	548226	**20.83%**
**inv.xml**	430107	1895850	1465743	**22.69%**
**vrt.xml**	97662	471683	374021	**20.71%**
**sprot41_1.xml**	332552	1763561	1431009	**18.86%**
**sprot41_2.xml**	441054	1966868	1525814	**22.42%**
**FullText Indexer2**	2655170	7521535	4866365	**35.30%**
**hum.xml**	144250	692476	548226	**20.83%**
**XML Import**	15859	25441	9582	**62.34%**
**TestGetPathways**	19658	21769	2111	**90.30%**
**TestBenchmark**	6840592	13531171	6690579	**50.55%**
**Totals (ms)**	**12229818**	**30934447**	**18704629**	**39.53%**
				
**Average of four measurements**				

	**FO Avg**	**KODO Avg**	**%(FO/KODO) Avg**	**FO/KODO CV**

**Junit Tests**	7875	15414	**51.11%**	2
**Boot**	3064	4319	**71.67%**	12
**SM Import**	402303	413655	**97.26%**	1
**FullText Indexer1**	572934	1818548	**31.51%**	1
**mam.xml**	59745	303455	**19.69%**	3
**rod.xml**	98938	429661	**23.02%**	3
**hum.xml**	152827	698456	**21.89%**	5
**inv.xml**	434253	1915082	**22.68%**	1
**vrt.xml**	100248	476812	**21.03%**	4
**sprot41_1.xml**	351278	1780467	**19.72%**	9
**sprot41_2.xml**	473765	1981667	**23.90%**	11
**FullText Indexer2**	2765967	7590177	**36.43%**	4
**XML Import**	16804	39913	**45.44%**	36
**TestGetPathways**	20388	24362	**84.19%**	10
**TestBenchmark**	6886598	13736330	**50.16%**	3
**Totals (ms)**	**12346986**	**31228318**	**39.54%**	
